# Investigation of PARP-1, PARP-2, and PARG interactomes by affinity-purification mass spectrometry

**DOI:** 10.1186/1477-5956-8-22

**Published:** 2010-04-13

**Authors:** Maxim Isabelle, Xavier Moreel, Jean-Philippe Gagné, Michèle Rouleau, Chantal Ethier, Pierre Gagné, Michael J Hendzel, Guy G Poirier

**Affiliations:** 1 Axe cancer, CHUQ Research Center, Faculty of Medicine, Laval University, 2705 Boulevard Laurier, Québec, Canada, G1V 4G2; 2 CNRS UMR 6061 Institut de Génétique et Développement de Rennes, Université de Rennes 1, IFR140, 2 Avenue du Pr Léon Bernard, Rennes, France; 3 Department of Oncology, University of Alberta and Cross Cancer Institute, Edmonton, Alberta, Canada, T6G 1Z2; 4 Proteomics Platform of the Quebec Genomics Center, Centre de recherche du CHUQ - CRCHUL, 2705 Boulevard Laurier, Québec, Canada, G1V 4G2

## Abstract

**Background:**

Poly(ADP-ribose) polymerases (PARPs) catalyze the formation of poly(ADP-ribose) (pADPr), a post-translational modification involved in several important biological processes, namely surveillance of genome integrity, cell cycle progression, initiation of the DNA damage response, apoptosis, and regulation of transcription. Poly(ADP-ribose) glycohydrolase (PARG), on the other hand, catabolizes pADPr and thereby accounts for the transient nature of poly(ADP-ribosyl)ation. Our investigation of the interactomes of PARP-1, PARP-2, and PARG by affinity-purification mass spectrometry (AP-MS) aimed, on the one hand, to confirm current knowledge on these interactomes and, on the other hand, to discover new protein partners which could offer insights into PARPs and PARG functions.

**Results:**

PARP-1, PARP-2, and PARG were immunoprecipitated from human cells, and pulled-down proteins were separated by gel electrophoresis prior to in-gel trypsin digestion. Peptides were identified by tandem mass spectrometry. Our AP-MS experiments resulted in the identifications of 179 interactions, 139 of which are novel interactions. Gene Ontology analysis of the identified protein interactors points to five biological processes in which PARP-1, PARP-2 and PARG may be involved: RNA metabolism for PARP-1, PARP-2 and PARG; DNA repair and apoptosis for PARP-1 and PARP-2; and glycolysis and cell cycle for PARP-1.

**Conclusions:**

This study reveals several novel protein partners for PARP-1, PARP-2 and PARG. It provides a global view of the interactomes of these proteins as well as a roadmap to establish the systems biology of poly(ADP-ribose) metabolism.

## Background

Poly(ADP-ribose) polymerases (PARPs) catalyze the formation of poly(ADP-ribose) (pADPr), a protein post-translational modification involved in several important biological processes, namely surveillance of genome integrity, cell cycle progression, initiation of the DNA damage response, apoptosis, and regulation of transcription (reviewed in [1]). Recently Kleine *et al. *[2] limited the PARP family to PARPs possessing the HYE catalytic core motif as well as a long β4/β5 loop, namely PARP-1, -2, -3, tankyrase-1 and -2, and vault-PARP. All other putative PARP family members were re-classified as mono-ADP-ribosyltransferases (PARP-6, -7, -8, -10, -11, -12, -14, -15, and -16) or catalytically inactive members (PARP-9 and -13). Poly(ADP-ribose) glycohydrolase (PARG), on the other hand, catabolizes pADPr and thereby accounts for the transient nature of poly(ADP-ribosyl)ation.

In this study, we chose to investigate PARP-1 and PARP-2 because of their pivotal role in the maintenance of genome integrity, and PARG to cover both the synthesis and degradation components of pADPr metabolism. Through their strand break-dependent PARP activity, both PARP-1 and PARP-2 are able to initiate a rapid response to DNA damage via pADPr synthesis on themselves (automodification) and on other nuclear acceptors such as histones. This DNA damage response facilitates base-excision repair (BER) [3,4] and contributes to non-homologous end joining (NHEJ) [5,6]. Empirical evidence however indicates that the functions of PARP-2 do not completely overlap those of PARP-1. Indeed, despite the significant PARP activity provided by PARP-2 in PARP-1 mouse knockout models following genotoxic stimulation [7,8], these knockouts present several phenotypes associated with genomic instability [9], demonstrating that PARP-2 cannot completely compensate for the loss of PARP-1. Furthermore, double PARP-1/PARP-2 mouse knockout is lethal at the embryonic stage, indicating that deficiency in DNA-dependent PARP activity cannot be functionally compensated for by PARP-3 or any other PARP family member, at least during early development [10]. The role of PARG is also vital as it is required for normal embryonic development and homeostatic cellular functions, and PARG-null embryos are not viable [11]. The interplay between PARPs and PARG, leading to marked shifts in the extent of poly(ADP-ribosyl)ation, is a temporally and spatially complex phenomenon as illustrated by the delocalization of PARP-1 from the nucleolus to the nucleoplasm following DNA damage [12] and the nucleocytoplasmic shuttling of PARG isoforms [13,14].

The PARPs-PARG system operates as a mechanism signaling DNA strand breaks, in which PARP-1 and PARP-2 play a dual role as damage sensors and signal transducers to several downstream effectors [15]. Automodified PARPs relay the signal to effector pathways by recruiting selected proteins into multiprotein complexes, which may then either directly participate in DNA repair or coordinate repair through chromatin unfolding [16]. For example, repair of single-strand breaks by BER involves a coordinated series of events wherein the protein XRCC1, recruited to the injured sites by PARP-1/2, operates as a scaffold that interacts with and stimulates the activity of enzymatic components of the BER machinery [17]. We have conducted a study aiming at the identification PARP-1/2 and PARG interactomes by AP-MS.

Our AP-MS protocol consisted in the following steps: A) The protein of interest (PARP-1, PARP-2, or PARG) was purified from a human cell lysate together with its binding partners. B) Proteins in the pulled-down complexes were separated by SDS-PAGE and then proteolyzed with trypsin. C) Tryptic peptides were analyzed by reverse-phase liquid chromatography followed by tandem mass spectrometry (LC-MS/MS) and D) database searching and statistical analysis were used to interpret the MS data and to yield the list of proteins that were present in the immunoprecipitates, including the bait protein, its interacting partners, and pulled-down contaminants. Our investigation of PARP-1, PARP-2, and PARG aimed to extend current knowledge on these proteins' interactomes by discovering new protein partners which could offer insights into PARPs and PARG functions.

## Results

### Identification of proteins associated with PARP-1, PARP-2 andPARG

**Figure 1:**
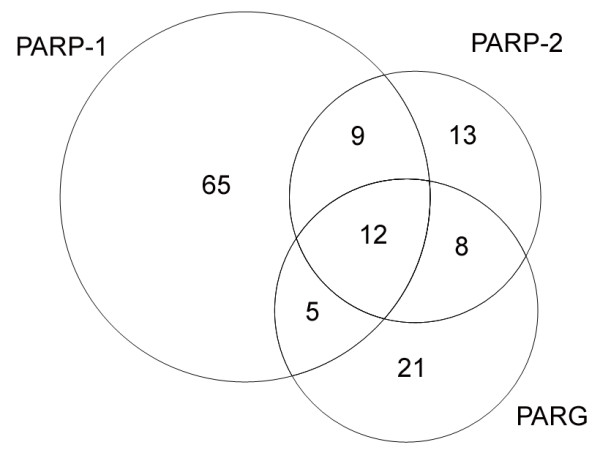
**Venn diagram illustrating the number of interactors identified by AP-MS for PARP-1, PARP-2 and PARG**. Numbers outside the overlaps correspond to proteins unique to either the PARP-1, PARP-2 or PARG immunoprecipitation dataset whereas numbers inside the overlaps correspond to proteins common to two or three datasets.

Our AP-MS experiments resulted in the identification of 133 protein interactors of PARP-1, PARP-2, and PARG. Additional file 1 lists these interactors and highlights whether or not they have been previously reported: green circles indicate previously reported interactors (a non-exhaustive list of references is provided) whereas red circles flag new interactors. We failed to observe some previously reported PARP-1, PARP-2, and PARG interactors; those are also included in Additional file 1 (blue circles) which thus summarizes both the findings of this study and current knowledge on the interactomes of these proteins. Ninety-one PARP-1 interactors, 42 PARP-2 interactors and 46 PARG interactors were identified; the total, 179, is higher than the number of identified proteins (133) because many interactors were pulled-down by more than one pADPr-metabolizing enzyme, as illustrated in Figure 1. PARP-1 had both the greatest number of interaction partners, 91, and of unique partners, 65. Additional file 2 synthesizes the immunoprecipitation data, namely the number of unique MS-identified peptides supporting the identification of the interactors and the protein sequence coverage corresponding to these peptides. Each of the 179 reported interactions was identified in at least two different pull-down assays wherein the interactor was identified by at least two high-confidence peptides (probability of correct identification ≥ 95%). In other words, Additional files 1 and 2 do not contain any single-peptide protein, nor any "one pull-down" protein, which were considered of lower confidence.

AP-MS led to the identification of novel interactions (Additional file 1, red circles): 69 for PARP-1, 37 for PARP-2, and 28 for PARG. Among the 179 interactions identified in this study, 42 have been previously reported (Additional file 1, green circles); most notable among these are KU70/KU80, DNA ligase III, DNA-dependent protein kinase (DNA-PK), Fragile × mental retardation 1 protein (FMR-1), and Nuclear factor kappa B subunit p50 (NFκBp50). These 42 interactions support the validity of our approach as many of these published interactions were detected using methodologies orthogonal to AP-MS. On the other hand, we did not detect 71 known protein interactors of PARP-1, PARP-2 or PARG (Additional file 1, blue circles), notably XRCC1 and DNA polymerase beta in the BER pathway, as well as TP53 and BCL-2 in the apoptosis pathway.

### Complementary immunoblot analyses

In order to check our affinity purification protocol, four previously reported interactors of PARP-1 and PARP-2 were probed by western blot: Nucleolin, RFC-1 (Replication factor C subunit 1), and NFκBp50 were probed in PARP-1 immunoprecipitates while PARP-1 was probed in a PARP-2 immunoprecipitate. All four proteins were detected using the antibody against the bait (PARP-1 or FLAG-PARP-2) but not detected in the control (data not shown), which supported the validity of our affinity purification protocol.

**Figure 2:**
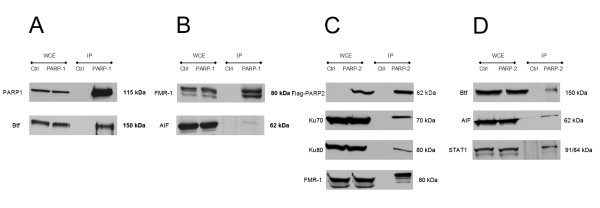
**Complementary western blot analysis of novel PARP-1 and PARP-2 interactors**. **A and B) **Immunoprecipitation of PARP-1 and associated proteins. In **A) **immunodetection of PARP-1 (bait) and the novel interactors Btf, identified by AP-MS. In **B) **immunodetection of novel PARP-1 interactors FMR-1 and AIF, absent from AP-MS. **C and D) **Immunoprecipitation of PARP-2 and associated proteins. In **A) **immunodetection of PARP-2 (bait) and the novel interactors KU70, KU80 and FMR-1, identified by AP-MS. In **B) **immunodetection of novel PARP-2 interactors BTF, AIF and STAT-1, absent from AP-MS. Each immunodetection is done in whole cellular extract (WCE) and immunoprecipitates (IP) from control (CTRL) and the bait (PARP-1 or FLAG-PARP-2).

Figure 2 presents eleven additional western blots, performed as a complement to AP-MS analysis. Two immunoblots verify that the bait was present in its immunoprecipitate as expected. It was indeed the case for both PARP-1 (Figure 2A) and FLAG-PARP-2 (Figure 2C). The remaining nine western blots were performed for new (unreported) protein interactors of PARP-1 and PARP-2. Firstly, four interactors that had been detected by AP-MS were probed: BTF (Bcl-2 associated transcription factor) in PARP-1 immunoprecipitate (Figure 2A); and Ku70, Ku80 and FMR-1 (Fragile × mental retardation protein 1) in FLAG-PARP-2 immunoprecipitates (Figure 2C). Secondly, five interactors absent from the AP-MS datasets were probed. These corresponded to interactions that were either hinted at by poor AP-MS data (low confidence identification) or of particular interest to our research group, namely the interactions between PARP-1 and either AIF (Apoptosis-inducing factor) or FMR-1 (Figure 2B), and between FLAG-PARP-2 and either BTF, AIF, or STAT1 (Signal transducer and activator of transcription 1) (Figure 2D). The presence of each the probed interactors was established by immunoblots, although the signal for AIF in the PARP-1 immunoprecipitate was very weak (Figure 2). These new interactions are reported as orange circles in Additional file 1. Finally, the presence of proteins was also established in the PARG immunoprecipitates confirming the findings of Gagné *et al*. [18] (data not shown).

### Functional analysis using Gene Ontology

**Figure 3:**
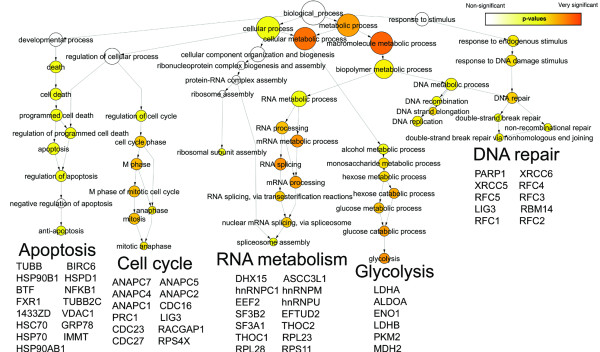
**Gene Ontology analysis of PARP-2 interactors**. Gene Ontology classification by biological process of the interactors of PARP-2 identified by AP-MS. See text for details.

**Figure 4:**
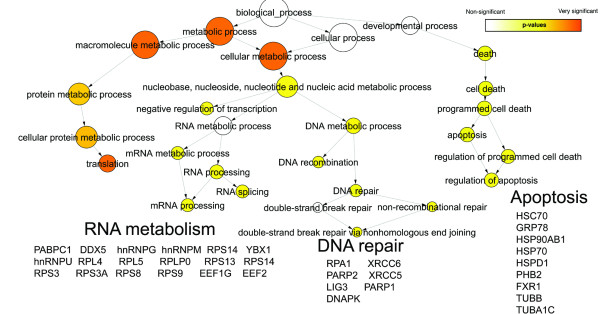
**Gene Ontology analysis of PARP-2 interactors**. Gene Ontology classification by biological process of the interactors of PARP-2 identified by AP-MS. See text for details.

**Figure 5:**
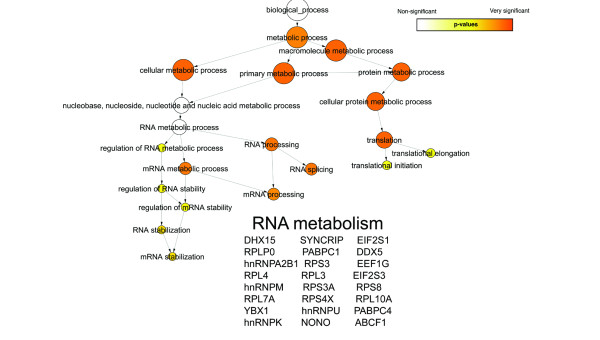
**Gene Ontology analysis of PARG interactors**. Gene Ontology classification by biological process of the interactors of PARG identified by AP-MS. See text for details.

The Gene Ontology (GO) project describes the cellular localizations, molecular functions, and biological processes of all annotated human proteins using a standardized vocabulary [19], and thus helps to cluster proteins into meaningful functional pathways and biological processes. Figures 3, 4, and 5 respectively depict the biological processes corresponding to the interactors of PARP-1, PARP-2 and PARG that we have observed by AP-MS (Additional file 1, green and red circles). Each figure illustrates a GO "hierarchical tree": the precise biological processes corresponding to the interactors appear as circles ("leaves") at the bottom of the graph and are connected to progressively broader parent biological processes as one goes up each "branch" towards the final biological process "trunk". Circles appearing in the graph are statistically over-represented biological process GO terms, as determined by the Biological Networks Gene Ontology tool (BiNGO). The size of each GO term's circle is proportional to the number of interactors corresponding to this term, and the color shading indicates the degree of statistical significance (a darker shade indicates higher significance). The protein interactors belonging to each branch are listed underneath it. For concision, only the abbreviations of the genes encoding these proteins are listed. These abbreviations precede the protein names in Additional file 1.

## Discussion

As previously mentioned, our investigation aimed to confirm current knowledge on the interactomes of PARP-1, PARP-2, and PARG as well as to discover new protein partners which could improve our understanding of into the functions of these key proteins. It was also hoped that a global view of the PARP-1, PARP-2 and PARG interactomes may help to grasp the ramifications of cancer treatment by PARP inhibitors, either in terms of therapeutic efficiency or side effects. Indeed, while PARP inhibitors have been recently shown to be active anticancer agents in BRCA1- and BRCA2-mutant tumors [20], fairly basic biological questions - including the biological roles of some of the interactors of PARP-1, PARP-2, and PARG - remain to be answered. Several PARP inhibitors are currently undergoing clinical trials and should be marketed as mid-stage results are promising (reviewed in [21]).

### Intrinsic strengths and limitations of AP-MS

Compared to yeast two-hybrid and related methods historically used to define protein interactomes, AP-MS has three major advantages. Firstly, it can be performed under near physiological conditions, in the relevant organism and cell type. Secondly, it does not typically affect post-translational modifications, which are often crucial for the organization and activity of complexes [22]. And thirdly, mass spectrometers can detect every abundant protein present in the immunoprecipitate, whether or not its presence is expected. Western blots, for instance, though very sensitive and specific, are incapable of that: they only target a few selected proteins expected to be in the sample from prior knowledge, and are therefore not conductive to the discovery of new, unanticipated protein interactors. Every analytical methodology - and AP-MS is no exception - possesses unique strengths and limitations, notably in terms of the number of different analytes it can detect simultaneously, and of its potential for false negatives and false positives.

#### False negatives, or "where did the interactors go?"

As pointed out by Gingras *et al*[22], high-throughput AP-MS datasets typically lack many previously documented protein-protein interactions. This was notably observed for two comprehensive AP-MS studies in yeast [23,24] and it is clearly the case of the current study's dataset as we failed to detect a number of known protein interaction of PARP-1, PARP-2 and PARG. Four factors likely contribute to this lack of detection [22]: 1) the proteins may not interact under the tested conditions; 2) when a bait is expressed with a tag, the nature and location of the tag may disrupt certain interactions; 3) the conditions of the AP bee too harsh to preserve interactions; and 4) the lack of detection may be due to a problem with the relative abundance of proteins in the AP sample. The latter typically occurs if the bait is in large molar excess over its interacting partners (a problem that is exacerbated when the bait is overexpressed, as in our case) or if the prey is in much lower abundance than other components of the sample (low stoichiometry). AP-MS thus tends to capture more stable and fairly abundant interactors, in contrast to other targeted approaches such yeast two-hybrid assays [22]. Finally interactions detected with western blots may not be detectable by AP-MS since western blot is generally more sensitive than MS for protein detection. We recognize that our investigation most probably missed certain low-abundant and/or low-affinity interactors of PARP-1, PARP-2 and PARG, and that more sensitive techniques will likely expand their interactomes in the future.

#### False positives, or "are the reported protein-proteininteractions real?"

Lack of specificity, i.e. reporting protein interactions that do not exist in the affinity-purified sample, may stem from either unspecific binding or incorrect protein identification. In the former case, immunoprecipitation material and reagents such as protein G, antibodies, or beads bind to proteins which are then falsely reported as binding the bait. In this study, two measures were taken to avoid reporting such non-specific interactions. Firstly, as detailed in Materials and Methods, BSA was added to the antibody-bead complex; BSA thus acted as a strong competitor (blocking agent) for all non-specific binding sites. Secondly, control immunoprecipitations using mouse immunoglobulin G as a bait were systematically used to discriminate between non-specific interactions and *bona fide *bait interactors. Proteins present in the controls were not reported. Reporting incorrectly identified proteins interactors was minimized through the use of stringent acceptance criteria for peptide and protein MS identifications, as described in Materials and Methods. Indeed, all reported peptide identifications have a probability of correct identification greater than 95%, and all reported protein identifications have a probability of correct identification greater than 99% and at least two accepted peptides. With these acceptance criteria, the overall MS false positive rate is estimated to be lower than 1%; in other words, the number of reported false positives due to incorrect protein identification should not exceed two. Mass spectrometry is widely recognized as a reliable method of identification of proteins in biological samples when the identifications are statistically validated and the false positive rate is kept low (reviewed in [25-27]). The use of Scaffold [28] to achieve such a statistical validation is also widely accepted with more than 40 recent peer-reviewed publications containing Scaffold-validated protein identifications. The fact that replicate AP-MS experiments were performed also increases confidence in the validity of the reported interactions as they were all observed twice or more.

#### Interpretation of the interaction datasets

A few points should be kept in mind while interpreting the PARP-1, PARP-2 and PARG datasets reported in Additional file 1. The first point is that AP-MS cannot distinguish between direct and indirect interactions; that is, reported interactions may correspond to two proteins which interact directly or, probably more frequently, correspond to two proteins which interact indirectly, via one or more bridging molecules (proteins, nucleic acids, or other molecules). The second point relates to overexpression of the bait. Transient over-expression of FLAG-PARP-2 and FLAG-PARG was required to circumvent the low specificity of available anti-PARP-2 and anti-PARG antibodies whereas FLAG-PARP-1 was transiently overexpressed for some of the PARP-1 experiments, in the hope of pulling some weak and/or scarce interactors of PARP-1 above detection limit. Such overexpressions may have resulted in the association of the bait protein with chaperones and could have led to improper intracellular localization [22]. Finally, one must keep in mind that some protein-protein interactions detected in a cell lysate may not actually occur *in vivo *if the two protein partners never co-localize within the cell.

### PARP-1 and PARP-2 may bind some proteins through their pADPr moiety

The PARP-1 and PARP-2 used as baits may have presented some degree of automodification, in which case their pADPr moiety - rather than their protein interaction domains - may have bound some protein interactors. Gagné *et al. *recently investigated the interactome of pADPr by AP-MS and reported a large number of pADPr-binding partners [29]. Interestingly, the interactors of PARP-1, PARP-2 and PARG listed in Additional file 2 include 53 of these pADPr-binding partners, and thus support the above hypothesis. These 53 common interactors are presented in Additional file 3 and consist mostly of DNA/RNA-binding proteins such as ribonucleoproteins, translation initiation factors, helicases, and ribosomal proteins.

### Functional analysis using Gene Ontology

BiNGO analysis suggests that PARP-1 may be involved in more biological processes than PARP-2 or PARG. Indeed, whereas all three enzymes are involved in RNA processing and both PARP-1 and PARP-2 participate in DNA repair, glycolysis and apoptosis, only PARP-1 partakes in cell cycle regulation and ubiquitin conjugation. The involvement of PARP-1 in DNA repair and apoptosis is well known (reviewed in [1]) and was expected. However, its apparent interaction with glycolysis and RNA metabolism proteins is surprising and may point to new PARP-1 functions. Devalaraja-Narashimha and Padanilam [30] recently studied the impact on glycolysis of the inhibition of glyceraldehyde phosphate-3-dehydrogenase by PARP-1, while others have focused on how NAD consumption (energy depletion) by PARP-1 influenced glycolysis [31-33]. PARP-1 is responsible for the majority of pADPr formation following DNA damage and excessive pADPr formation promotes shuttling of AIF to the nucleus and caspase-independent cell death [34]. The detailed mechanism is yet to be understood, but it has been suggested that pADPr may be shuttled from the nucleus to the cytoplasm via proteins, resulting in AIF translocation, as well as the sequestration of anti-apoptotic proteins or the activation of pro-apoptotic proteins [35,36]. The "apoptosis" branch of Figure 4 suggests that PARP-2 probably also plays a role in apoptosis, presumably because it can also add pADPr to proteins. PARP-2 apparently plays a less important role in apoptosis than PARP-1 given the lower complexity of PARP-2's apoptosis branch. The biological processes shown for PARG in Figure 5 are mainly linked to RNA metabolism and confirm the findings of previous studies. Indeed, with the majority of PARG activity located in the cytoplasm, maintaining tight spatial regulation of this enzyme depends on nucleocytoplasmic shuttling proteins such as the ribonucleoprotein (RNP) complexes [18]. Moreover, poly(ADP-ribosyl)ation of heterogenous nuclear ribonucleoproteins (hnRNPs) was determined to be of importance for protein splicing in Drosophila, suggesting that PARP-1 and PARG modulate splicing pathways through regulation of interactions between hnRNPs and RNA [37].

### Involvement of PARP-1, PARP-2 and PARG in RNA metabolism

Figures 3, 4 and 5 indicate that PARP-1, PARP-2 and PARG are all involved in RNA metabolism and, more precisely, in RNA splicing. For instance, hnRNP M and hnRNP U, which are both part of the spliceosome C complex [38], are among the 11 pulled-down proteins common to PARP-1, PARP-2 and PARG. Moreover, PARP-1, PARP-2 and PARG also interact with several other members of this complex (Additional file 1). PARP-1 interacts with splicing factor 3A1, splicing factor 3B2, splicing factor 3B1, hnRNP C, snRNP EFTUD2 and U5 snRNP 200 kDa; PARP-2 with DEAD box polypeptide 48, ATP-dependent RNA helicase DDX5, hnRNP A1, hnRNP G and Polyadenylate-binding protein 1; and PARG interacts with ATP-dependent RNA helicase DDX5, hnRNP A1, hnRNP Q, hnRNP A2B1 and Polyadenylate-binding protein 1. Many of these proteins share interactions with more than one pADPr-metabolizing enzyme. The fact that each bait pulled down different components of the spliceosome C complex might be explained by the dynamic nature of this complex but is more probably indicative of a lack of affinity for the bait, or of a lack of analytical sensitivity.

Our dataset includes other interesting PARP-1 interactors related to transcription and splicing, namely the THO/TREX and FMRP complexes. THO complex subunits 1, 2, 5 and 6 were identified by mass spectrometry in PARP-1 immunoprecipitates. THO/TREX is required for the nuclear export of mRNA and is coupled to transcriptional elongation [39,40]. The THO complex is composed of multiple subunits linked to Aly and Bat1, which form the TREX complex responsible for RNA export. The THO/TREX complex is also recruited to splice mRNA, and is indirectly associated to transcription via the splicing machinery [41]. Many components of the FMRP complex, which suppresses translation of selected mRNA within large mRNP complexes [42], are present in our dataset: Fragile-X mental retardation syndrome-related protein 1 (FXR1) (common to PARP-1, PARP-2 and PARG datasets); FXR2 and FMR1 (PARP-2 and PARG); and nuclear Fragile-X mental retardation-interacting protein 2 (PARG only). While PARP-1 [43] and PARG [18] have already been identified in mRNP complexes, their role therein remains unclear although some evidence pointed toward pADPr regulation of transcriptional activity. Our finding that PARP-1 and PARP-2 are associated with FMRP particles strengthens this hypothesis. Both PARP-2 and PARG also interact with nuclease-sensitive element-binding protein 1 (YBX-1), which is involved in mRNA binding, regulation of mRNA stability, translation efficiency and, possibly, binding splicing enhancer elements [44,45].

The involvement of PARP-1, PARP-2 and PARG in the regulation of RNA splicing is corroborated by many studies. Recently, Ji and Tulin [37] demonstrated in Drosophila that endogenous PARP-1 and PARG regulate pADPr binding to hnRNPs, subsequently altering the RNA-binding ability of hnRNPs and modulating splicing. Another study by Malanga *et al. *[46] confirmed that pADPr can bind the splicing factor ASF/SF2 within domains crucial for splicing activity, thereby regulating splicing. pADPr is also involved in transcription-splicing through its role in Cajal bodies [47]. Small spliceosomal components first accumulate in Cajal bodies and then undergo a spliceosome phase assembly; they are then transported to the cytoplasm for splicing events and are finally reintegrated to Cajal bodies to be recycled [48,49]. Automodified PARP-1 is responsible for protein shuttling into Cajal bodies and PARP-1 is itself crucial for the integrity of Cajal bodies [47]. Furthermore, PARG deletion leads to an imbalance of proteins in the Cajal bodies, demonstrating that pADPr is an important regulator of shuttling into Cajal bodies. As illustrated above, PARP-1, PARP-2 and PARG share several interactors related to splicing and transcription, and poly(ADP-ribosyl)ation, or pADPr binding, presumably underlies these interactions. Indeed, and as mentioned previously, many interactors of PARP-1, PARP-2 and PARG identified in this study are also known to be associated with pADPr [29]. One can hypothesize that PARP-1, PARP-2 and PARG regulate some transcriptional and splicing events through the addition and subsequent removal of pADPr chains on specific substrates.

### Dual DNA-dependent roles of PARP-1 and PARP-2: DNA damage signaling and cell death

It is well known that genomic stress induces pADPr formation via DNA-dependent PARP activation. As the severity of DNA strand breaks increases, more pADPr is synthesized and the extent of poly(ADP-ribosyl)ation determines the cellular response: mild genotoxic stress induces PARP-1 and PARP-2 activation and signaling to promote DNA repair while severe stress generates extreme pADPr accumulation that triggers cell death.

#### DNA damage signaling

BiNGO analysis revealed similar significance for the "DNA repair" branches of PARP-1 and PARP-2 (Figure 3 and 4). Both PARP-1 and PARP-2 immunoprecipitates contained KU70 and KU80, two proteins involved in DNA double-strand break repair by NHEJ, thus confirming the redundant function of PARP-1 and PARP-2 in the surveillance of genome integrity. Other interactors linked to the DNA repair function of PARP-1 and PARP-2 are RNA-binding protein 14 [50] (observed for both PARPs) and Replication factor C (RFC) subunits 1, 2, 3, 4 and 5 (observed for PARP-1 only). RFC1, RFC2, RFC3, RFC4 and RFC5 form a heteropentamer complex that interacts with PCNA and enables the binding of its N-terminal DNA-binding domain to duplex DNA. This mechanism is essential in the recognition of non-primer template DNA structures during replication and/or repair [51]. PARP-2 interactors also include Replication Factor A protein 1 and the catalytic subunit of DNA-dependent protein kinase (DNA-PK). The former protein participates in the very early stages of initiation in both DNA recombination and DNA replication, through association with DNA-PK and, possibly, through the recruitment of NHEJ proteins [52]. Globally, our interactor datasets are consistent with the known roles of PARP-1 and PARP-2 in DNA repair initiation via the BER [4,53,54] and NHEJ [5,55] pathways. Our results suggest that PARP-1 and PARP-2 may signal DNA damage by means of multiple complexes in order to speed up DNA repair.

#### Cell death

As previously mentioned, activation of the DNA-dependent PARP-1 and PARP-2 under severe genomic stress leads to substantial accumulation of pADPr, which triggers AIF translocation, and eventually cell death [34]. Our study reveals some unknown details of this apoptotic pathway. For instance, GRP78, HSC70, HSP90AB1, and HSP70, four heat shock proteins, are all interactors of PARP-1, PARP-2, and PARG. Although heat shock proteins are frequent immunoprecipitation contaminants [56], the above four proteins were absent from our control datasets and we consequently believe them to be *bona fide *interactors. These heat shock proteins act as chaperones and are likely to attach to multiple unrelated proteins, given that they bind mutated and unfolded proteins to prevent their secretion. However, some heat shock proteins exhibit interactions or functions similar to PARP-1 and PARP-2, notably in apoptosis. For instance, GRP78 is involved in the negative regulation of apoptosis by suppressing the activation of caspase-7 and caspase-12 [57] while HSC70, an anti-apoptotic co-chaperone, inhibits HSP90 and other apoptotic proteins [58]. HSP90AB1 interacts with TP53 within a multi-chaperone complex in which HSP70 plays an important role in the process of apoptosis [59]. The heat shock protein 60kDa mitochondrial (HSP60) is another interactor that is shared by PARP-1 and PARP-2. One of the known functions of HSP60 is its contribution to the regulation of apoptosis through its association with caspase-3 [60] and BAX [61]. Also of interest among PARP-1 interactors are Baculoviral IAP repeat-containing protein 6 (BIRC6) and BTF. The latter binds to DNA and represses transcription of survival genes [62], while BIRC6 is an inhibitor of apoptosis through its inhibition of caspases, particularly of caspase-3 [63].

An interesting hypothesis yet to be confirmed is that poly(ADP-ribosyl)ation affects multiple proteins in order to shift the balance between pro-apoptotic and anti-apoptotic molecules. Indeed, interfering with NAD and pADPr metabolism increases mRNA and protein GRP78 levels [64,65]. *Parp-1*^-/- ^fibroblast cells exhibit increased expression of HSP70 [66], a protein that delays nuclear translocation of AIF [67]. Moreover, increased levels of HSP70 reversibly inactivate PARG, and thus cause pADPr accumulation [66]. Our observation of likely protein interactions between PARP-1/2 and some heat shock proteins raises the possibility that these PARPs could affect the function of heat shock proteins during apoptosis through transcriptional regulation.

### A possible new function for PARP-1

The role of pADPr in the cell cycle is generally associated with mitotic spindle functions. This structure consists of an array of microtubules and various molecules that self-organize to align and then segregate chromosomes during mitosis. pADPr is found at the spindle and is required for its function [68]. Our data highlights that another possible role of PARP-1 in the cell cycle, as the regulation of the APC/C complex. Indeed, we report here for the first time that PARP-1 interacts with eight of the twelve proteins belonging to the APC/C complex, one of the two poly-ubiquitylating E3 ligase complexes influencing cell cycle progression. APC/C is largely associated with cell cycle progression and sister chromatid separation (reviewed in [69]). The four APC/C proteins not observed in PARP-1 immunoprecipitates have low molecular weights (21 kDa and lower) and are therefore more challenging to identify by mass spectrometry since they generate fewer tryptic peptides upon digestion. This is probably why they were not detected. Chang *et al. *[68] demonstrated that PARP-1 is not critical for mitotic progression and that it does not play an essential role in the regulation of anaphase entry in the cell cycle. However, PARP-1 has been shown to bind and poly(ADP-ribosyl)ate BUB3 [70], which is essential for the recruitment of this protein to centromeric heterochromatin [71]. BUB3 is suggested to act as a regulator of the APC/C complex. It is unclear how PARP-1 could be involved in mitotic progression, but our findings motivate further investigation of this potential function.

## Conclusion

Affinity-purification mass spectrometry enabled us to confirm and extend current knowledge on the interactomes of PARP-1, PARP-2 and PARG, and provided a global view of pADPr metabolism and of its various functional ramifications. This study confirms the participation of DNA-dependent PARPs in complexes related to multiple DNA damage pathways and suggests that PARP-1 and PARP-2 may partake in other processes, notably RNA metabolism (PARP-1, PARP-2) and the regulation of the APC/C complex (PARP-1). Interactions between PARG and multiple proteins involved in RNA metabolism seem also highly likely. While building effective mechanistic models covering all the functions of PARP-1, PARP-2 and PARG will be a long and arduous process, defining their interactomes is clearly the first step. Our results complement the list of pADPr-binding proteins and pADPr-associated proteins published by Gagne *et al. *[29]. Taken together, these datasets provide the groundwork for a system-wide modeling of the effects of poly(ADP-ribosyl)ation on biological processes.

## Materials and methods

### Cell culture

Human neuroblastoma SK-N-SH and HeLa cervical carcinoma cell lines were cultured (5% CO2, 37°C) in DMEM medium supplemented with 10% foetal bovine serum (Hyclone-ThermoFisher Scientific, Canada). Penicillin (100 U/ml) and streptomycin (100 mg/ml) (Wisent, Canada) were added to culture media.

### Immunoprecipitation and immunoblotting

Cells were grown at 80-90% confluence in 150 mm culture dishes then washed with ice-cold phosphate-buffered saline (PBS). Ice-cold phosphate lysis buffer (175 mM KPO4, pH 8.0, 150 mM NaCl, 1% NP-40, 1 mM DTT, 0.5 mM PMSF, and Complete protease-inhibitor cocktail, according to Roche diagnostics' instructions) or Tris lysis buffer (175 mM Tris, pH 8.0, 150 mM NaCl, 1% NP-40, 1 mM DTT, 0.5 mM PMSF, and Complete protease-inhibitor cocktail) was added to cells. Cells were harvested using a cell scraper. Lysed cells collected from three dishes were pooled, then gently mixed by inversion for 1 hour at 4°C, and centrifuged for 10 min at 6000 × g to remove insoluble cellular debris. The cellular extract was mixed with magnetic beads coupled to protein G (Dynal, Invitrogen, Canada) and the appropriate monoclonal antibody (F1-23 for PARP-1, M2 for FLAG-PARP-2 and FLAG-PARG), or normal mouse IgG for the control, and incubated 2 hours at 4°C with rotation. The beads had been previously blocked for 1 hour with 1% BSA and washed with the appropriate lysis buffer. Proteins were eluted from the beads by boiling for 5 min in Laemmli SDS sample buffer containing 5% (v/v) β-mercaptoethanol.

Eluted proteins were separated by SDS-PAGE and then transferred onto 0.2 μm nitrocellulose membrane (Bio-Rad, Canada). After one hour incubation with a blocking solution (PBS-T containing 5% non-fat milk), the membrane was probed overnight at 21°C with shaking, with either: primary antibodies to PARP-1 [clone C2-10 (1:5000)], Apoptosis-inducing factor (AIF) [(1:5000), Sigma, USA], ATP-dependent DNA helicase II 80 kDa subunit (Ku80) [(1:5000), Oncogene Research Products, USA], ATP-dependent DNA helicase II 70 kDa subunit (Ku 70) [(1:5000), Oncogene Research Products, USA], Bcl-2-associated transcription factor 1 (Btf) [(1:1000), BD Pharmingen, USA], M2 [(1:1000), BD Pharmingen, USA], Fragile-X mental retardation 1 protein (FMR-1) [(1:5000), Chemica, USA], or Signal transducer and activator of transcription 1-alpha/beta (STAT-1) [(1:2500), Cell Signalling, USA]. After washing with PBS-T (PBS containing 0.1% Tween-20), membrane were incubated for 1 hour with species-specific horseradish peroxidase-conjugated secondary antibody. Signals were detected with the Western Lightning Chemiluminescence reagent plus kit (Perkin Elmer, USA).

### LC-MS/MS analysis

Proteins eluted from the immunoprecipitated material were separated by SDS-PAGE. The gel was then stained with SYPRO Ruby fluorescent protein stain (Bio-Rad, Canada) as per the manufacturer's instructions. The entire protein profile on the gel was sliced into 30 sections using a gel excision Lanepicker (The Gel Company, USA). In-gel protein digests were performed on a MassPrep liquid handling station (Micromass, USA), according to the manufacturer's instructions, using sequencing-grade modified trypsin (Promega, USA). Peptide extracts were dried out, resuspended in 10 μl of 0.1% formic acid in water, and analyzed by LC-MS/MS using either an ion trap (LCQ Deca XP or LTQ, Thermo Fisher, Canada) or a QqTOF (QStar XL, MDS Analytical Technologies, Canada) mass spectrometer.

#### Ion trap analysis

Peptides were separated by reverse-phase liquid chromatography and eluted into the spectrometer via a nanoelectrospray (nanoESI) ion source (all equipment from Thermo Fisher). Peptide chromatography was achieved with a BioBasic C18 PicoFrit column (75 μm ID × 10 cm, New Objective, USA) at a flow rate of 200 nL/min with a 30-min linear gradient from 2 to 50% buffer B (acetonitrile with 0.1% formic acid) against buffer A (water with 0.1% formic acid). Mass spectra were acquired using a data-dependent acquisition mode whereby each MS-only scan (400 to 2000 m/z) was followed by collision-induced dissociation (CID) of either the three (LCQ) or seven (LTQ) most intense ions. Dynamic exclusion was set to 30 sec ND relative collisional fragmentation energy to 35%.

#### qQTOF analysis

LC-MS/MS was performed using an Agilent 1100 nanoLC system coupled to a QSTAR XL equipped with MDS's nanoESI source. Peptides were separated using the aforesaid New Objective column running at 200 nL/min, with a 25-min linear separation gradient from 2% to 25% B followed by a 10-min linear gradient from 25% to 40% B 10 min (buffers A and B as above). Mass spectra were acquired using a data-dependent acquisition mode whereby each MS-only scan (400 to 1800 m/z) was followed by CID of the three most intense ions having a +2, +3 or +4 charge. Fragmented precursors were dynamically excluded for 60 seconds with a 100-ppm mass tolerance.

### Interpretation of MS/MS spectra and acceptance criteria for peptide and protein identifications

Ion trap MS/MS spectra were analyzed using Mascot [72] (version 2.2.04, Matrix Science, UK) and SEQUEST (version SRF v.2, Thermo Electron, USA) [73] whereas the QqTOF MS/MS spectra were analyzed using Mascot and X!Tandem (version TORNADO 2008.02.01.3, http://www.thegpm.org) [74] which are more appropriate for high mass accuracy datasets. SEQUEST, Mascot and X!Tandem were set up to search the human IPI HUMAN database (version 3.42, 72149 entries) assuming a digestion with trypsin. Search parameters were as follows: fragment ion mass tolerance: 0.5 Da (Mascot, X!Tandem) or 2.0 Da (SEQUEST); parent ion tolerance: 2.0 Da (all search engines); fixed modification: carbamidomethylation of cysteine (all); variable modifications: oxidation of methionine (all), deamidation of asparagines and glutamine, and acetylation of lysine and arginine (Mascot and X!Tandem only). Two missed cleavages were allowed.

Scaffold (version 2.2.1, Proteome Software, USA) was used to validate MS/MS-based peptide and protein identifications. Peptide identifications were accepted if they could be established at greater than 95.0% probability as specified by the Peptide Prophet algorithm [75]. Protein identifications were accepted if they could be established at greater than 99.0% probability and contained at least 2 identified peptides. Protein probabilities were assigned by the Protein Prophet algorithm [76]. Proteins that contained similar peptides and could not be differentiated based on MS/MS analysis alone were grouped to satisfy the principles of parsimony. Using these stringent identification parameters, the rate of false positive identifications is less than 1%. Furthermore, any protein not identified in at least two independent immunoprecipitation experiments was discarded.

### Clustering of identified proteins by biological processes

Proteins were classified as per the biological processes ontology of the Gene Ontology (GO) [77] and hierarchical graphs of overrepresented GO terms were created using BiNGO v2.0 [78] and Cytoscape v2.5 [79]. GO annotations p-values were computed using the hypergeometric statistical test (cluster versus the whole annotation bank) and corrected with BiNGO's Benjamini and Hochberg False Discovery Rate feature.

## Competing interests

The authors declare that they have no competing interests.

## Authors' contributions

MI and GGP conceived, designed, and coordinated the study, as well as drafted the manuscript. XM, JPG, MR and CE participated in sample preparation and performed the immunoprecipitations and western blot analyses. MI and PG carried out mass spectrometry, bioinformatics, and statistical analysis. GGP and MHJ provided direction and funding for this project. All authors read and approved the manuscript.

## Supplementary Material

Additional file 1 PDF Table, Proteins interactors of PARP-1, PARP-2 and PARG2. Click here for file

Additional file 2 Excel spreadsheet, Immunoprecipitation data for each interactor identified by affinity-purification mass spectrometry (AP-MS). Click here for file

Additional file 3 Excel spreadsheet, Known poly(ADP-ribose)-binding proteins (or acceptors of poly(ADP-ribosyl)ation modification) that were immunoprecipitated with either PARP-1, PARP-2 or PARG. Click here for file
