# Supplementary material for: Investigation of PARP-1, PARP-2, and PARG interactomes by affinity-purification mass spectrometry
Source: Proteome Sci. 2010 Apr 13;8:22. doi: 10.1186/1477-5956-8-22 (PMC2861645; doi:10.1186/1477-5956-8-22)
Supplement: Additional file 1 — PDF Table, Proteins interactors of PARP-1, PARP-2 and PARG2. [file 1477-5956-8-22-S1.PDF]

Supplementary Table 1. Proteins interactors of PARP-1, PARP-2, and PARG. Supplementary Table 1 summarizes both this study's findings and previously reported interactors of PARP-1, PARP-2, and PARG. Interactors are divided into broad biological process categories and presents, for each interactor, the protein's UniProt accession code and name, as well as colored circles indicating who identified it as an interactor of either PARP-1, PARP-2 or PARG. Interactors identified by affinity-purification mass spectrometry (AP-MS) in this study are flagged by a <u>red circle</u> when they have not been reported (new interactors) and by a <u>green circle</u> when they have, in which case a non-exhaustive list of references is provided. Previously reported PARP-1, PARP-2, and PARG interactors that were not observed in this study are shown as <u>blue circles</u>, with an accompanying non-exhaustive list of references. New, unreported interactors identified solely by western blot are shown as <u>orange circles</u>. Each of the AP-MS interactions (red and green circles) reported meets the criteria listed in the Methods section.

Uniprot Code / Gene name	Protein name	PARP-1	PARP-2	PARG	Ref.
**Cell cycle**					
Q9H1A4 ANAPC1	Anaphase-promoting complex subunit 1	red	empty	empty	-/-/-
Q9UJX6 ANAPC2	Isoform 1 of Anaphase-promoting complex subunit 2	red	empty	empty	-/-/-
Q9UJX5 ANAPC4	Isoform 1 of Anaphase-promoting complex subunit 4	red	empty	empty	-/-/-
Q9UJX4 ANAPC5	Isoform 1 of Anaphase-promoting complex subunit 5	red	empty	empty	-/-/-
Q9UJX3 ANAPC7	Anaphase-promoting complex subunit 7	red	empty	empty	-/-/-
P50990 CCT8	59 kDa protein	empty	red	empty	-/-/-
Q13042 CDC16	Isoform 1 of Cell division cycle protein 16 homolog	red	empty	empty	-/-/-
Q9UJX2 CDC23	Cell division cycle protein 23	red	empty	empty	-/-/-
P30260 CDC27	Cell division cycle protein 27 homolog	red	empty	empty	-/-/-
O43663 PRC1	Isoform 1 of Protein regulator of cytokinesis 1	red	empty	empty	-/-/-
Q9H0H5 RACGAP1	Rac GTPase-activating protein 1	red	empty	empty	-/-/-
P62701 RPS4X	40S ribosomal protein S4, X isoform	red	red	empty	-/-/-
P49450 CENPA	Centromeric protein A	blue	blue	empty	[5]/ [5]/-
P07199 CENPB	Centromeric protein B	blue	blue	empty	[5]/ [5]/-
O43684 BUB3	Mitotic checkpoint protein Bub3	blue	blue	empty	[5]/ [5]/-
Q15843 NEDD8	Ubiquitin-like protein Nedd8	blue	empty	empty	[6]/-/-
**Cell Death**					
Q9NYF8 BCLAF1	Isoform 1 of Bcl-2-associated transcription factor 1	red	orange	empty	-/-/-
Q9NR09 BIRC6	Baculoviral IAP repeat-containing 6	red	empty	empty	-/-/-
P07900 HSP90AA1	Heat shock protein 90kDa alpha (cytosolic), class A member 1 isoform 1	empty	red	empty	-/-/-
P14625 HSP90B1	Endoplasmin	red	red	empty	-/-/-
P08238 HSP90AB1	Heat shock protein HSP 90-beta	red	red	red	-/-/-
P08107 HSPA1B;HSPA1A	Heat shock 70 kDa protein 1	green	red	green	[1]/-/[2]
P11021 HSPA5	HSPA5 protein	red	red	red	-/-/-
P11142 HSPA8	Isoform 1 of Heat shock cognate 71 kDa protein	red	red	green	-/-/[2]

Uniprot Code / Gene name	Protein name	PARP-1	PARP-2	PARG	Ref.
**Cell Death (continued)**					
P10809 HSPD1	60 kDa heat shock protein, mitochondrial	● red	● red	○	-/-/-
Q16891 IMMT	Isoform 1 of Mitochondrial inner membrane protein	● green	○	○	[3]/-/-
P19838 NFKB1	Isoform 2 of Nuclear factor NF-kappa-B p105 subunit	● green	○	○	[4]/-/-
P07437 TUBB	Tubulin beta chain	● red	● red	○	-/-/-
P68371 TUBB2C	Tubulin beta-2C chain	● red	○	● red	-/-/-
P21796 VDAC1	Voltage-dependent anion-selective channel protein 1	● red	○	○	-/-/-
P63104 YWHAZ	14-3-3 protein zeta/delta	● red	○	○	-/-/-
O95831 AIF	Apoptosis inducing factor 1	● orange	● orange	○	-/-/-
P42574 CASP3	Caspase 3	● blue	○	● blue	[7]/-/[8]
P04637 TP53	Cellular tumor antigen p53	● blue	○	○	[11, 12]/-/-
P10415 BCL2	Apoptosis regulator Bcl2	● blue	○	○	[14]/-/-
Q04206 NFKB3	Nuclear factor NF-kappa-B p65 subunit	● blue	○	○	[4]/-/-
P29466 CASP1	Caspase 1	● blue	○	○	[17, 18]/-/-
P55210 CASP7	Caspase 7	● blue	○	○	[20]/-/-
Q14790 CASP8	Caspase 8	○	● blue	○	-/[21]/-

Uniprot Code / Gene name	Protein name	PARP-1	PARP-2	PARG	Ref.
**DNA Repair**					
P78527 PRKDC	DNA-dependent protein kinase catalytic subunit	● blue	● green	○	[22]/[23] /-
Q15233 NONO	Non-POU domain-containing octamer-binding protein	○	○	● red	-/-/-
Q86W56 PARG	Poly(ADP-ribose) glycohydrolase	● blue	○	● green	[24, 25]/-/-
P09874 PARP1	Poly(ADP-ribose) polymerase 1	● green	● green	● green	[27]/[5]/[25]
Q9UGN5 PARP2	Isoform 2 of Poly(ADP-ribose) polymerase 2	● blue	● green	○	[5]/-/-
Q96PK6 RBM14	Isoform 1 of RNA-binding protein 14	● green	○	● red	[29]/-/-
P13010 XRCC5	ATP-dependent DNA helicase 2 subunit 2 Ku80	● green	● red	○	[30, 31]/-/-
P12956 XRCC6	ATP-dependent DNA helicase 2 subunit 1 Ku70	● green	● red	○	[30, 32]/-/-
P49916 LIG3	Ligase III, DNA ATP-dependent isoform beta precursor	● green	● green	○	[33]/[15]/-
Q9Y6F1 PARP3	poly(ADP-ribose) polymerase 3	● blue	○	○	[9, 10]/-/-
Q13315 ATM	Ataxia telangiectasia mutated	● blue	○	○	[13]/-/-
P18887 XRCC1	X-ray repair croos-complementing protein 1	● blue	● blue	○	[15]/[15]/-
Q7Z2E3 APTX	Aprataxin	● blue	○	○	[16]/-/-
Q03468 ERCC6	DNA excision repair protein	● blue	○	○	[19]/-/-

DNA Replication & Transcription

Uniprot Code / Gene name	Protein name	PARP-1	PARP-2	PARG	Ref.
P13639 EEF2	Elongation factor 2	🔴	⚪	⚪	-/-/-
P05198 EIF2S1	Eukaryotic translation initiation factor 2 subunit 1	⚪	⚪	🔴	-/-/-
P41091 EIF2S3	Eukaryotic translation initiation factor 2 subunit 3	⚪	⚪	🔴	-/-/-
P16402 HIST1H1D	Histone H1.3	⚪	⚪	🔴	-/-/-
P62805 HIST4H4	Histone H4	🟢	⚪	⚪	[26]/-/-
Q71DI3 HIST2H3D	Histone H3.2	🔴	⚪	⚪	-/-/-
P35251 RFC1	Isoform 1 of Replication factor C subunit 1	🟢	⚪	⚪	[28]/-/-
P35250 RFC2	Isoform 1 of Replication factor C subunit 2	🟢	⚪	⚪	[28]/-/-
P40938 RFC3	Replication factor C subunit 3	🟢	⚪	⚪	[28]/-/-
P35249 RFC4	Replication factor C subunit 4	🟢	⚪	⚪	[28]/-/-
P40937 RFC5	Replication factor C subunit 5	🟢	⚪	⚪	[28]/-/-
Q6IQ15 EEF1A1	Elongation factor 1-alpha	🔴	🔴	🔴	-/-/-
P27694 RPA1	Replication protein A 70 kDa DNA-binding subunit	⚪	🔴	⚪	-/-/-
P26641 EEF1G	Elongation factor 1-gamma	⚪	⚪	🔴	-/-/-
P19793 RXRA	Retinoid X receptor, alpha	🔵	⚪	⚪	[38]/-/-
P06748 NPM1	Nucleophosmin /B23	🔵	🔵	⚪	[39, 40]/[40]/-
P14859 POU2F1	Octamer binding transcription factor 1	🔵	⚪	⚪	[41]/-/-
P10276 RARA	Retinoic acid receptor alpha	🔵	⚪	⚪	[42]/-/-
P09884 POLA1	DNA polymerase, alpha	🔵	⚪	⚪	[43]/-/-
P10242 MYB	myb proto-oncogene protein	🔵	⚪	⚪	[45, 46]/-/-
P11388 TOP2A	DNA topoisomerase II alpha	🔵	⚪	⚪	[48]/-/-
Q9HAN9 NMNAT1	Nicotinamide mononucleotide adenylyltransferase 1	🔵	⚪	⚪	[50]/-/-
Q09472 P300/CBP	Histone acetyl transferase p300	🔵	⚪	⚪	[53]/-/-
Q13547 HDAC1	Histone deacetylase 1	🔵	⚪	⚪	[53]/-/-
P35269 TFIIF	General transcription factor IIF subunit RAP74	🔵	⚪	⚪	[57]/-/-
P06746 POLB	DNA polymerase beta	🔵	🔵	⚪	[15]/[15]/-
Q02880 TOP2B	DNA topoisomerase II beta	🔵	⚪	⚪	[59]/-/-
P11387 TOP1	DNA topoisomerase I	🔵	⚪	⚪	[60]/-/-
P12004 PCNA	Proliferating cell nuclear antigen	🔵	⚪	⚪	[61]/-/-
Q16656 NRF-1	Nuclear respiratory factor 1	🔵	⚪	⚪	[34]/-/-
Q9Y618 SMRT	thyroid hormone receptor	🔵	⚪	⚪	[35]/-/-
P09429 HMGB1	High mobility group protein 1	🔵	⚪	⚪	[36, 37]/-/-
P52926 HMGA2	High mobility group protein 2	🔵	⚪	⚪	[37]/-/-
Q6FI13 HISTH2A	Histone H2A	🔵	🔵	⚪	[26]/[15]/-
P62807 HISTH2B	Histone H2B	🔵	🔵	⚪	[26]/[15]/-

Uniprot Code / Gene name	Protein name	PARP-1	PARP-2	PARG	Ref.
**DNA Replication & Transcription (continued)**					
P07305 HISTH1	Histone H1	● (blue)	● (blue)	○	[26]/[15]/-
P49715 CEBPA	C/EBPalpha	● (blue)	○	○	[44]/-/-
P28347 TEF-1	Transcriptional enhancer factor TEF-1	● (blue)	○	○	[47]/-/-
Q9NYB0 TRF2	Telomeric repeat-bining factor 2	○	● (blue)	○	-/[49]/-
Q01094 E2F1	Retinoblastoma-binding protein 3 RBBP-3	● (blue)	○	○	[51, 52]/-/-
Q14191 WRN	Werner syndrome ATP-dependent helicase	● (blue)	○	○	[54]/-/-
P05549 AP-2	Transcription factor AP-2	● (blue)	○	○	[55, 56]/-/-
Q15361 TTF-1	Transcription termination factor 1	● (blue)	● (blue)	○	[58]/[58]/-
**Glycolysis & Energy metabolism**					
P06576 ATP5B	ATP synthase subunit beta, mitochondrial	○	● (red)	○	-/-/-
P06733 ENO1	Isoform alpha-enolase of Alpha-enolase	● (red)	○	○	-/-/-
P00338 LDHA	Isoform 1 of L-lactate dehydrogenase A chain	● (red)	○	○	-/-/-
P07195 LDHB	L-lactate dehydrogenase B chain	● (red)	○	○	-/-/-
P40926 MDH2	Malate dehydrogenase, mitochondrial	● (red)	○	○	-/-/-
P04075 ALDOA	Fructose-bisphosphate aldolase A	● (red)	○	○	-/-/-
P14618 PKM2	Isoform M2 of Pyruvate kinase isozymes M1/M2	● (red)	○	○	-/-/-

Uniprot Code / Gene name	Protein name	PARP-1	PARP-2	PARG	Ref.
**RNA metabolism**					
O00571 DDX3X	ATP-dependent RNA helicase DDX3X	○	○	● (red)	-/-/-
Q86XP3 DDX42	Isoform 1 of ATP-dependent RNA helicase DDX42	● (red)	○	○	-/-/-
P17844 DDX5	Probable ATP-dependent RNA helicase DDX5	○	● (red)	● (red)	-/-/-
Q9BQ39 DDX50	ATP-dependent RNA helicase DDX50	● (red)	○	○	-/-/-
O43143 DHX15	Putative pre-mRNA-splicing factor ATP-dependent RNA helicase DHX15	● (green)	○	● (red)	[62]/-/-
Q08211 DHX9	ATP-dependent RNA helicase A	● (red)	○	● (red)	-/-/-
Q15029 EFTUD2	116 kDa U5 small nuclear ribonucleoprotein component	● (green)	○	○	[62]/-/-
Q06787 FMR1	Fragile X mental retardation 1 protein	● (orange)	● (red)	● (green)	-/-/[2]
P51114 FXR1	Fragile X mental retardation syndrome-related protein 1	● (red)	● (red)	● (green)	-/-/[2]
P51116 FXR2	Fragile X mental retardation syndrome-related protein 2	○	● (red)	● (green)	-/-/[2]
P09651 HNRNPA1	Isoform A1-B of Heterogeneous nuclear ribonucleoprotein A1	○	○	● (green)	-/-/[2]
P22626 HNRNPA2B1	Isoform B1 of Heterogeneous nuclear ribonucleoproteins A2/B1	○	○	● (green)	-/-/[2]
P07910 HNRNPC	Heterogeneous nuclear ribonucleoproteins C1/C2	● (green)	○	○	[62]/-/-
P61978 HNRNPK	Heterogeneous nuclear ribonucleoprotein K	● (blue)	○	● (red)	[62]/-/-
P52272 HNRNPM	Isoform 1 of Heterogeneous nuclear ribonucleoprotein M	● (red)	● (red)	● (green)	-/-/[2]
Q00839 HNRNPU	Heterogeneous nuclear ribonucleoprotein U	● (blue)	● (red)	● (green)	[62]/-/[2]
Q9Y6M1 IGF2BP2	Insulin-like growth factor 2 mRNA binding protein 2	○	● (red)	○	-/-/-

Uniprot Code / Gene name	Protein name	PARP-1	PARP-2	PARG	Ref.
**RNA metabolism (continued)**					
Q12905 ILF2	Interleukin enhancer-binding factor 2	○	red	○	-/-/-
Q12906 ILF3	Isoform 5 of Interleukin enhancer-binding factor 3	○	○	red	-/-/-
Q6ZS99 NCL	Nucleolin	green	blue	green	[39]/[15]/[2]
Q7Z417 NUFIP2	Nuclear fragile X mental retardation-interacting protein 2	○	○	green	-/-/[2]
P11940 PABPC1	Isoform 1 of Polyadenylate-binding protein 1	○	red	green	-/-/-
Q13310 PABPC4	Isoform 1 of Polyadenylate-binding protein 4	○	○	red	-/-/-
P38159 RBMX	Heterogeneous nuclear ribonucleoprotein G	○	red	○	-/-/-
P46777 RPL5	60S ribosomal protein L5	○	red	○	-/-/-
P62424 RPL7A	60S ribosomal protein L7a	○	○	red	-/-/-
P05388 RPLP0	60S acidic ribosomal protein P0	○	red	green	-/-/[2]
P62906 RPL10A	60S ribosomal protein L10a	○	○	red	-/-/-
P50914 RPL14	60S ribosomal protein L14	○	○	red	-/-/-
P62829 RPL23	60S ribosomal protein L23	red	○	○	-/-/-
P46779 RPL28	60S ribosomal protein L28	red	○	○	-/-/-
P39023 RPL3	60S ribosomal protein L3	○	○	red	-/-/-
P36578 RPL4	60S ribosomal protein L4	○	red	green	-/-/[2]
O75533 SF3B1	Splicing factor 3B subunit 1	green	○	○	[62]/-/-
Q13435 SF3B2	splicing factor 3B subunit 2	red	○	○	-/-/-
O60506 SYNCRIP	Isoform 1 of Heterogeneous nuclear ribonucleoprotein Q	○	○	red	-/-/-
P62241 RPS8	40S ribosomal protein S8	○	red	red	-/-/-
P46781 RPS9	40S ribosomal protein S9	○	red	○	-/-/-
P19474 TRIM21	52 kDa Ro protein	red	red	○	-/-/-
P67809 YBX1	Nuclease-sensitive element-binding protein 1	○	red	green	-/-/[2]
Q15459 SF3A1	Splicing factor 3 subunit 1	red	○	○	-/-/-
P62280 RPS11	40S ribosomal protein S11	red	○	○	-/-/-
P62277 RPS13	40S ribosomal protein S13	red	red	○	-/-/-
P62263 RPS14	40S ribosomal protein S14	red	red	○	-/-/-
P62249 RPS16	40S ribosomal protein S16	red	○	○	-/-/-
P62269 RPS18;LOC100130553	40S ribosomal protein S18	red	○	○	-/-/-
P62851 RPS25	40S ribosomal protein S25	red	○	○	-/-/-
P23396 RPS3	40S ribosomal protein S3	red	red	red	-/-/-
P61247 RPS3A	40S ribosomal protein S3a	green	red	red	[14]/-/-
Q96FV9 THOC1	THO complex subunit 1	red	○	○	-/-/-

Uniprot Code / Gene name	Protein name	PARP-1	PARP-2	PARG	Ref.
**RNA metabolism (continued)**					
Q8NI27 THOC2	THO complex 2 isoform 1	●	○	○	-/-/-
Q13769 THOC5	THO complex subunit 5 homolog	●	○	○	-/-/-
Q86W42 THOC6	THO complex subunit 6 homolog	●	○	○	-/-/-
Q5TAX3 ZCCHC11	Isoform 1 of Zinc finger CCHC domain-containing protein 11	●	○	○	-/-/-
Q8NE71 ABCF1	Isoform 2 of ATP-binding cassette sub-family F member 1	○	○	●	-/-/-
O75643 ASCC3L1	Isoform 1 of U5 small nuclear ribonucleoprotein 200 kDa helicase	● (green)	○	○	[62]/-/-
Q14444 CAPRIN1	Isoform 1 of Caprin-1	○	○	●	-/-/-
Q6PI48 DARS2	Aspartyl-tRNA synthetase, mitochondrial	●	○	○	-/-/-

Uniprot Code / Gene name	Protein name	PARP-1	PARP-2	PARG	Ref.
**Others**					
A8K9N6 AHSG	Alpha-2-HS-glycoprotein	●	○	○	-/-/-
P07355 ANXA2	Annexin A2 isoform 1	●	○	○	-/-/-
Q9NX63 CHCHD3	Coiled-coil-helix-coiled-coil-helix domain-containing protein 3, mitochondrial	●	○	○	-/-/-
Q14247 CTTN	Src substrate cortactin	●	○	○	-/-/-
O75165 DNAJC13	homolog subfamily C, member 13	○	○	●	-/-/-
Q14739 LBR	Lamin-B receptor	●	○	○	-/-/-
Q86V48 LUZP1	Isoform 1 of Leucine zipper protein 1	●	○	○	-/-/-
Q15365 PCBP1	Poly(rC)-binding protein 1	●	○	○	-/-/-
P07737 PFN1	Profilin-1	●	○	○	-/-/-
Q9Y512 SAMM50	Sorting and assembly machinery component 50 homolog	●	○	○	-/-/-
Q9H267 VPS33B	Vacuolar protein sorting-associated protein 33B	●	○	○	-/-/-
Q9Y2W1 THRAP3	Thyroid hormone receptor-associated protein 3	●	○	○	-/-/-
Q9UPN9 TRIM33	Isoform Beta of E3 ubiquitin-protein ligase TRIM33	●	○	○	-/-/-
Q71U36 TUBA1A	Tubulin alpha-1A chain	●	○	○	-/-/-
Q9BQE3 TUBA1C	Tubulin alpha-1C chain	○	●	○	-/-/-
P45880 VDAC2	Voltage-dependent anion-selective channel protein 2	●	○	○	-/-/-

Uniprot Code / Gene name	Protein name	PARP-1	PARP-2	PARG	Ref.
**Others (continued)**					
O15014 ZNF609	Zinc finger protein 609	● (red)	○	○	-/-/-
P42224 STAT-1	Signal transducer and activator of transcription 1-alpha/beta	○	● (orange)	○	-/-/-
P37231 PPARG	Perosisome proliferators-activated receptor gamma	○	● (blue)	○	-/[63]/-
Q9UH65 SWP70	Switch associated protein 70	● (blue)	○	○	[39]/-/-
P08311 CTSG	Cathepsin G	● (blue)	○	○	[64]/-/-
P10144 GZMB	Granzyme B	● (blue)	○	○	[65]/-/-
P54368 OAZ	Ornithine decarboxylase antizyme	● (blue)	○	○	[66]/-/-
P28482 ERK1/2	Mitogen activated protein kinase	● (blue)	○	○	[67]/-/-
P45983 JNK1	c-Jun N-terminal kinase 1	● (blue)	○	○	[68]/-/-
Q9H2K8 hKFC	serine/threonine-protein kinase	● (blue)	○	○	[69]/-/-
P35222 CTNB	Beta Catenin	● (blue)	○	○	[70]/-/-

Uniprot Code / Gene name	Protein name	PARP-1	PARP-2	PARG	Ref.

References:

1. Kotoglou, P., et al., *Hsp70 translocates to the nuclei and nucleoli, binds to XRCC1 and PARP-1, and protects HeLa cells from single-strand DNA breaks.* Cell Stress Chaperones, 2009. **14**(4): p. 391-406.
2. Gagne, J.P., et al., *Poly(ADP-ribose) glycohydrolase is a component of the FMRP-associated messenger ribonucleoparticles.* Biochem J, 2005. **392**(Pt 3): p. 499-509.
3. Rossi, M.N., et al., *Mitochondrial localization of PARP-1 requires interaction with mitofilin and is involved in the maintenance of mitochondrial DNA integrity*. J Biol Chem, 2009. **284**(46): p. 31616-24.

4. Hassa, P.O. and M.O. Hottiger, *The functional role of poly(ADP-ribose)polymerase 1 as novel coactivator of NF-kappaB in inflammatory disorders.* Cell Mol Life Sci, 2002. **59**(9): p. 1534-53.
5. Saxena, A., et al., *Poly(ADP-ribose) polymerase 2 localizes to mammalian active centromeres and interacts with PARP-1, Cenpa, Cenpb and Bub3, but not Cenpc.* Hum Mol Genet, 2002. **11**(19): p. 2319-29.
6. Li, T., et al., *A general approach for investigating enzymatic pathways and substrates for ubiquitin-like modifiers.* Arch Biochem Biophys, 2006. **453**(1): p. 70-4.
7. D'Amours, D., et al., *Proteolysis of poly(ADP-ribose) polymerase by caspase 3: kinetics of cleavage of mono(ADP-ribosyl)ated and DNA-bound substrates.* Radiat Res, 1998. **150**(1): p. 3-10.
8. Affar, E.B., et al., *Caspase-3-mediated processing of poly(ADP-ribose) glycohydrolase during apoptosis.* J Biol Chem, 2001. **276**(4): p. 2935-42.
9. Augustin, A., et al., *PARP-3 localizes preferentially to the daughter centriole and interferes with the G1/S cell cycle progression.* J Cell Sci, 2003. **116**(Pt 8): p. 1551-62.
10. Rouleau, M., et al., *PARP-3 associates with polycomb group bodies and with components of the DNA damage repair machinery.* J Cell Biochem, 2007. **100**(2): p. 385-401.
11. Malanga, M., et al., *Poly(ADP-ribose) binds to specific domains of p53 and alters its DNA binding functions.* J Biol Chem, 1998. **273**(19): p. 11839-43.
12. Wesierska-Gadek, J., J. Wojciechowski, and G. Schmid, *Phosphorylation regulates the interaction and complex formation between wt p53 protein and PARP-1.* J Cell Biochem, 2003. **89**(6): p. 1260-84.
13. Aguilar-Quesada, R., et al., *Interaction between ATM and PARP-1 in response to DNA damage and sensitization of ATM deficient cells through PARP inhibition.* BMC Mol Biol, 2007. **8**: p. 29.
14. Song, D., S. Sakamoto, and T. Taniguchi, *Inhibition of poly(ADP-ribose) polymerase activity by Bcl-2 in association with the ribosomal protein S3a.* Biochemistry, 2002. **41**(3): p. 929-34.
15. Schreiber, V., et al., *Poly(ADP-ribose) polymerase-2 (PARP-2) is required for efficient base excision DNA repair in association with PARP-1 and XRCC1.* J Biol Chem, 2002. **277**(25): p. 23028-36.
16. Gueven, N., et al., *Aprataxin, a novel protein that protects against genotoxic stress.* Hum Mol Genet, 2004. **13**(10): p. 1081-93.
17. Lazebnik, Y.A., et al., *Cleavage of poly(ADP-ribose) polymerase by a proteinase with properties like ICE.* Nature, 1994. **371**(6495): p. 346-7.
18. Gu, Y., et al., *Cleavage of poly(ADP-ribose) polymerase by interleukin-1 beta converting enzyme and its homologs TX and Nedd-2.* J Biol Chem, 1995. **270**(32): p. 18715-8.
19. Thorslund, T., et al., *Cooperation of the Cockayne syndrome group B protein and poly(ADP-ribose) polymerase 1 in the response to oxidative stress.* Mol Cell Biol, 2005. **25**(17): p. 7625-36.
20. Germain, M., et al., *Cleavage of automodified poly(ADP-ribose) polymerase during apoptosis. Evidence for involvement of caspase-7.* J Biol Chem, 1999. **274**(40): p. 28379-84.
21. Benchoua, A., et al., *Active caspase-8 translocates into the nucleus of apoptotic cells to inactivate poly(ADP-ribose) polymerase-2.* J Biol Chem, 2002. **277**(37): p. 34217-22.
22. Ruscetti, T., et al., *Stimulation of the DNA-dependent protein kinase by poly(ADP-ribose) polymerase.* J Biol Chem, 1998. **273**(23): p. 14461-7.

23. Tramontano, F., S. Di Meglio, and P. Quesada, *Co-localization of poly(ADPR)polymerase 1 (PARP-1) poly(ADPR)polymerase 2 (PARP-2) and related proteins in rat testis nuclear matrix defined by chemical cross-linking.* J Cell Biochem, 2005. **94**(1): p. 58-66.
24. Keil, C., T. Grobe, and S.L. Oei, *MNNG-induced cell death is controlled by interactions between PARP-1, poly(ADP-ribose) glycohydrolase, and XRCC1.* J Biol Chem, 2006. **281**(45): p. 34394-405.
25. Keil, C., E. Petermann, and S.L. Oei, *Tannins elevate the level of poly(ADP-ribose) in HeLa cell extracts.* Arch Biochem Biophys, 2004. **425**(1): p. 115-21.
26. D'Amours, D., et al., *Poly(ADP-ribosyl)ation reactions in the regulation of nuclear functions.* Biochem J, 1999. **342 (Pt 2)**: p. 249-68.
27. Pion, E., et al., *DNA-induced dimerization of poly(ADP-ribose) polymerase-1 triggers its activation.* Biochemistry, 2005. **44**(44): p. 14670-81.
28. Droit, A., et al., *PARPs database: a LIMS systems for protein-protein interaction data mining or laboratory information management system.* BMC Bioinformatics, 2007. **8**: p. 483.
29. Iwasaki, T., W.W. Chin, and L. Ko, *Identification and characterization of RRM-containing coactivator activator (CoAA) as TRBP-interacting protein, and its splice variant as a coactivator modulator (CoAM).* J Biol Chem, 2001. **276**(36): p. 33375-83.
30. Li, B., et al., *Identification and biochemical characterization of a Werner's syndrome protein complex with Ku70/80 and poly(ADP-ribose) polymerase-1.* J Biol Chem, 2004. **279**(14): p. 13659-67.
31. Galande, S. and T. Kohwi-Shigematsu, *Caught in the act: binding of Ku and PARP to MARs reveals novel aspects of their functional interaction.* Crit Rev Eukaryot Gene Expr, 2000. **10**(1): p. 63-72.
32. Idogawa, M., et al., *Ku70 and poly(ADP-ribose) polymerase-1 competitively regulate beta-catenin and T-cell factor-4-mediated gene transactivation: possible linkage of DNA damage recognition and Wnt signaling.* Cancer Res, 2007. **67**(3): p. 911-8.
33. Leppard, J.B., et al., *Physical and functional interaction between DNA ligase IIIalpha and poly(ADP-Ribose) polymerase 1 in DNA single-strand break repair.* Mol Cell Biol, 2003. **23**(16): p. 5919-27.
34. Hossain, M.B., et al., *Poly(ADP-ribose) Polymerase 1 Interacts with Nuclear Respiratory Factor 1 (NRF-1) and Plays a Role in NRF-1 Transcriptional Regulation.* J Biol Chem, 2009. **284**(13): p. 8621-32.
35. Jeyakumar, M., et al., *Phosphorylation of thyroid hormone receptor-associated nuclear receptor corepressor holocomplex by the DNA-dependent protein kinase enhances its histone deacetylase activity.* J Biol Chem, 2007. **282**(13): p. 9312-22.
36. Ditsworth, D., W.X. Zong, and C.B. Thompson, *Activation of poly(ADP)-ribose polymerase (PARP-1) induces release of the pro-inflammatory mediator HMGB1 from the nucleus.* J Biol Chem, 2007. **282**(24): p. 17845-54.
37. Tanuma, S., T. Yagi, and G.S. Johnson, *Endogenous ADP ribosylation of high mobility group proteins 1 and 2 and histone H1 following DNA damage in intact cells.* Arch Biochem Biophys, 1985. **237**(1): p. 38-42.
38. Miyamoto, T., T. Kakizawa, and K. Hashizume, *Inhibition of nuclear receptor signalling by poly(ADP-ribose) polymerase.* Mol Cell Biol, 1999. **19**(4): p. 2644-9.
39. Borggrefe, T., et al., *A B-cell-specific DNA recombination complex.* J Biol Chem, 1998. **273**(27): p. 17025-35.
40. Meder, V.S., et al., *PARP-1 and PARP-2 interact with nucleophosmin/B23 and accumulate in transcriptionally active nucleoli.* J Cell Sci, 2005. **118**(Pt 1): p. 211-22.
41. Nie, J., et al., *Interaction of Oct-1 and automodification domain of poly(ADP-ribose) synthetase.* FEBS Lett, 1998. **424**(1-2): p. 27-32.
42. Pavri, R., et al., *PARP-1 determines specificity in a retinoid signaling pathway via direct modulation of mediator.* Mol Cell, 2005. **18**(1): p. 83-96.

43. Dantzer, F., et al., *Functional association of poly(ADP-ribose) polymerase with DNA polymerase alpha-primase complex: a link between DNA strand break detection and DNA replication.* Nucleic Acids Res, 1998. **26**(8): p. 1891-8.
44. Yin, H. and J. Glass, *In prostate cancer cells the interaction of C/EBPalpha with Ku70, Ku80, and poly(ADP-ribose) polymerase-1 increases sensitivity to DNA damage.* J Biol Chem, 2006. **281**(17): p. 11496-505.
45. Cervellera, M.N. and A. Sala, *Poly(ADP-ribose) polymerase is a B-MYB coactivator.* J Biol Chem, 2000. **275**(14): p. 10692-6.
46. Santilli, G., et al., *PARP co-activates B-MYB through enhanced phosphorylation at cyclin/cdk2 sites.* Oncogene, 2001. **20**(57): p. 8167-74.
47. Butler, A.J. and C.P. Ordahl, *Poly(ADP-ribose) polymerase binds with transcription enhancer factor 1 to MCAT1 elements to regulate muscle-specific transcription.* Mol Cell Biol, 1999. **19**(1): p. 296-306.
48. Yusufzai, T.M., et al., *CTCF tethers an insulator to subnuclear sites, suggesting shared insulator mechanisms across species.* Mol Cell, 2004. **13**(2): p. 291-8.
49. Dantzer, F., et al., *Functional interaction between poly(ADP-Ribose) polymerase 2 (PARP-2) and TRF2: PARP activity negatively regulates TRF2.* Mol Cell Biol, 2004. **24**(4): p. 1595-607.
50. Berger, F., C. Lau, and M. Ziegler, *Regulation of poly(ADP-ribose) polymerase 1 activity by the phosphorylation state of the nuclear NAD biosynthetic enzyme NMN adenylyl transferase 1.* Proc Natl Acad Sci U S A, 2007. **104**(10): p. 3765-70.
51. Simbulan-Rosenthal, C.M., et al., *PARP-1 binds E2F-1 independently of its DNA binding and catalytic domains, and acts as a novel coactivator of E2F-1-mediated transcription during re-entry of quiescent cells into S phase.* Oncogene, 2003. **22**(52): p. 8460-71.
52. Simbulan-Rosenthal, C.M., et al., *Poly(ADP-ribose) polymerase upregulates E2F-1 promoter activity and DNA pol alpha expression during early S phase.* Oncogene, 1999. **18**(36): p. 5015-23.
53. Hassa, P.O., et al., *Acetylation of poly(ADP-ribose) polymerase-1 by p300/CREB-binding protein regulates coactivation of NF-kappaB-dependent transcription.* J Biol Chem, 2005. **280**(49): p. 40450-64.
54. von Kobbe, C., et al., *Poly(ADP-ribose) polymerase 1 regulates both the exonuclease and helicase activities of the Werner syndrome protein.* Nucleic Acids Res, 2004. **32**(13): p. 4003-14.
55. Kannan, P., et al., *PolyADP-ribose polymerase is a coactivator for AP-2-mediated transcriptional activation.* Nucleic Acids Res, 1999. **27**(3): p. 866-74.
56. Li, M., et al., *Dual regulation of AP-2alpha transcriptional activation by poly(ADP-ribose) polymerase-1.* Biochem J, 2004. **382**(Pt 1): p. 323-9.
57. Rawling, J.M. and R. Alvarez-Gonzalez, *TFIIF, a basal eukaryotic transcription factor, is a substrate for poly(ADP-ribosyl)ation.* Biochem J, 1997. **324 (Pt 1)**: p. 249-53.
58. Maeda, Y., et al., *PARP-2 interacts with TTF-1 and regulates expression of surfactant protein-B.* J Biol Chem, 2006. **281**(14): p. 9600-6.
59. Ju, B.G. and M.G. Rosenfeld, *A breaking strategy for topoisomerase IIbeta/PARP-1-dependent regulated transcription.* Cell Cycle, 2006. **5**(22): p. 2557-60.
60. Malanga, M. and F.R. Althaus, *Poly(ADP-ribose) reactivates stalled DNA topoisomerase I and Induces DNA strand break resealing.* J Biol Chem, 2004. **279**(7): p. 5244-8.
61. Frouin, I., et al., *Human proliferating cell nuclear antigen, poly(ADP-ribose) polymerase-1, and p21waf1/cip1. A dynamic exchange of partners.* J Biol Chem, 2003. **278**(41): p. 39265-8.
62. Czubaty, A., et al., *Proteomic analysis of complexes formed by human topoisomerase I.* Biochim Biophys Acta, 2005. **1749**(1): p. 133-41.

63. Bai, P., et al., *Poly(ADP-ribose) polymerase-2 [corrected] controls adipocyte differentiation and adipose tissue function through the regulation of the activity of the retinoid X receptor/peroxisome proliferator-activated receptor-gamma [corrected] heterodimer.* J Biol Chem, 2007. **282**(52): p. 37738-46.
64. Gobeil, S., et al., *Characterization of the necrotic cleavage of poly(ADP-ribose) polymerase (PARP-1): implication of lysosomal proteases.* Cell Death Differ, 2001. **8**(6): p. 588-94.
65. Andrade, F., et al., *Granzyme B directly and efficiently cleaves several downstream caspase substrates: implications for CTL-induced apoptosis.* Immunity, 1998. **8**(4): p. 451-60.
66. Ku, M.C., S. Stewart, and A. Hata, *Poly(ADP-ribose) polymerase 1 interacts with OAZ and regulates BMP-target genes.* Biochem Biophys Res Commun, 2003. **311**(3): p. 702-7.
67. Kauppinen, T.M., et al., *Direct phosphorylation and regulation of poly(ADP-ribose) polymerase-1 by extracellular signal-regulated kinases 1/2.* Proc Natl Acad Sci U S A, 2006. **103**(18): p. 7136-41.
68. Zhang, S., et al., *c-Jun N-terminal kinase mediates hydrogen peroxide-induced cell death via sustained poly(ADP-ribose) polymerase-1 activation.* Cell Death Differ, 2007. **14**(5): p. 1001-10.
69. Gwack, Y., et al., *Poly(ADP-ribose) polymerase 1 and Ste20-like kinase hKFC act as transcriptional repressors for gamma-2 herpesvirus lytic replication.* Mol Cell Biol, 2003. **23**(22): p. 8282-94.
70. Idogawa, M., et al., *Poly(ADP-ribose) polymerase-1 is a component of the oncogenic T-cell factor-4/beta-catenin complex.* Gastroenterology, 2005. **128**(7): p. 1919-36.
